# DUX4 expressing immortalized FSHD lymphoblastoid cells express genes elevated in FSHD muscle biopsies, correlating with the early stages of inflammation

**DOI:** 10.1093/hmg/ddaa053

**Published:** 2020-04-02

**Authors:** Christopher R S Banerji, Maryna Panamarova, Peter S Zammit

**Affiliations:** King's College London, Randall Centre for Cell and Molecular Biophysics, New Hunt's House, Guy's Campus, London SE1 1UL, UK

## Abstract

Facioscapulohumeral muscular dystrophy (FSHD) is an incurable disorder linked to ectopic expression of *DUX4*. However, DUX4 is notoriously difficult to detect in FSHD muscle cells, while DUX4 target gene expression is an inconsistent biomarker for FSHD skeletal muscle biopsies, displaying efficacy only on pathologically inflamed samples. Immune gene misregulation occurs in FSHD muscle, with DUX4 target genes enriched for those associated with inflammatory processes. However, there lacks an assessment of the FSHD immune cell transcriptome, and its contribution to gene expression in FSHD muscle biopsies. Here, we show that EBV-immortalized FSHD lymphoblastoid cell lines express *DUX4* and both early and late DUX4 target genes. Moreover, a biomarker of 237 up-regulated genes derived from FSHD lymphoblastoid cell lines is elevated in FSHD muscle biopsies compared to controls. The FSHD Lymphoblast score is unaltered between FSHD myoblasts/myotubes and their controls however, implying a non-myogenic cell source in muscle biopsies. Indeed, the FSHD Lymphoblast score correlates with the early stages of muscle inflammation identified by histological analysis on muscle biopsies, while our two late DUX4 target gene expression biomarkers associate with macroscopic inflammation detectable via MRI. Thus, FSHD lymphoblastoid cell lines express *DUX4* and early and late DUX4 target genes, therefore, muscle-infiltrated immune cells may contribute the molecular landscape of FSHD muscle biopsies.

## Introduction

Facioscapulohumeral muscular dystrophy (FSHD) is a prevalent [12/100 000 (1)] inherited disorder. Clinically, FSHD manifests as a skeletal muscle dystrophy, typically commencing in the facial muscles before progressing to the shoulder girdle and muscles of the lower limb (2,3). The pattern of muscle involvement in FSHD is also often left/right asymmetric (4). Heterogeneity in clinical progression between first-degree relatives, including monozygotic twins, is also well described (5–7). Extra-muscular features including retinal telangiectasia similar to Coat’s disease (8–10) and sensorineural hearing loss (11,12) in some patients suggest a more systemic distribution in FSHD pathology.

FSHD shows an autosomal dominant pattern of inheritance linked to epigenetic derepression of the D4Z4 macrosatellite at chromosome 4q35 (13,14). This epigenetic modification can be achieved by two non-mutually exclusive genomic events: either deletion of D4Z4 units to leave 1–10 repeats on at least one chromosome 4 as occurs in FSHD1 (MIM 158900) (>95% of FSHD cases) (15–17), and/or mutations in the chromatin-modifying gene *SMCHD1* (18)*,* or more rarely *DNMT3B* (19), in FSHD2 (MIM 158901). In addition to epigenetic derepression at D4Z4, FSHD patients also carry a permissive 4qA haplotype encoding a poly(A) signal in the flanking pLAM region (13). Each 3.3 kb D4Z4 unit contains an open reading frame for a retrogene coding for the transcription factor double homeobox 4 (DUX4) (20,21). Epigenetic derepression at D4Z4 permits expression of *DUX4* transcripts from the distal-most D4Z4 unit, which are then stabilized by the poly(A) signal in non-coding exon 3 (22). Crucially, at least one D4Z4 unit is required for FSHD (23). Misexpression of DUX4 protein is thus proposed to underlie pathology in both FSHD1 and FSHD2 (13).

How DUX4 drives pathology in FSHD is poorly understood. DUX4 induces a set of genes in myoblasts that are pro-apoptotic and anti-myogenic (24–28), but curiously also immune system related (29,30). However, detection of DUX4 in FSHD patient muscle biopsies and derived myogenic cultures is notoriously difficult, with *DUX4* expression reported to be as low as in 1/1000–1/5000 myoblasts and 1/200 myotube nuclei (29,31). DUX4 target gene expression is proposed as a biomarker for FSHD muscle biopsies (30), but we have demonstrated via meta-analysis that its discriminatory power is generally underwhelming (32). However, appreciable levels of DUX4 target genes are detectable in muscle biopsies that have been preselected for active disease/inflammation via magnetic resonance imaging (MRI) metrics of T1 and Short-TI Inversion Recovery (STIR) positivity (33). Given this, we investigated other biomarkers for FSHD muscle biopsies. The homeodomains of DUX4 show homology with the homeodomain of the myogenic master regulator PAX7, and a competitive interaction has been shown between DUX4 and PAX7 proteins (27,34). The PAX7 homeodomain can also substitute those of DUX4 without affecting certain functions of DUX4 (34). We demonstrated that a biomarker based on suppression of PAX7 target genes hallmarks FSHD muscle biopsies, as well as isolated myoblasts, significantly outperforming DUX4 target gene expression (32,33).

PAX7 target gene repression and DUX4 target gene activation, however, independently associate with the degree of histological inflammation and active disease in MRI-guided FSHD muscle biopsies, implying that while both target gene sets contribute to pathology, there are potentially multiple pathomechanisms (32). Given that *DUX4* is expressed at such low levels in patient muscle cells, the question remains as to which cells are expressing *DUX4* and its target genes in these highly inflamed biopsies? Histological evidence of muscle inflammation in FSHD is well documented (2,35–38) with perivascular (predominantly CD4^+^) and endomysial (mainly CD8^+^) lymphocytic infiltrates a consistent finding, which is clear in STIR-positive muscle biopsies. Furthermore, elevated levels of circulating pro-inflammatory cytokines in FSHD such as TNFα are inversely associated with maximal voluntary contraction in quadriceps (39).

DUX4 induces expression of immune system-related genes in myoblasts (29) and inflammatory genes are dysregulated in FSHD muscle biopsies (30,40). Recently, a library has been characterized of 114 FSHD and control Epstein-Barr virus (EBV)-immortalized B-lymphoblastoid cell lines (LCLs) from 12 FSHD1 affected families (41,42). The degree of demethylation at D4Z4 in the FSHD LCL clones is as expected for FSHD1, and the 61 FSHD LCLs generally display robust *DUX4* expression, as well as DUX4 target genes *ZSCAN4*, *TRIM43* and *MBD3L2* (42). Curiously, a small number of control LCLs also express DUX4, albeit at significantly lower levels to FSHD LCLs (42). Of further relevance, a significant subset of B-cell acute lymphoblastic leukaemia (B-ALL) cases present with a hybrid DUX4-IGH fusion gene in which the N-terminus-located homeodomains of DUX4 are fused to a clamp-like transactivation domain of IGH (43,44). DUX4-IGH can arrest B-cell differentiation and induce transformation (43,44).

*DUX4* expression in FSHD patient-derived immune cells may represent a non-myogenic contributor to pathology and associate with the elevated levels of DUX4 target genes in inflamed FSHD muscle biopsies. Here, we performed RNA-seq of FSHD and control LCLs and primary myoblasts and myotubes to analyze *DUX4*, early and late DUX4 target gene expressions and to generate an FSHD lymphoblast biomarker. All three FSHD LCL lines expressed *DUX4* on RNA-seq, compared with no detectible *DUX4* transcripts in 18 FSHD myoblast, and in 15/18 FSHD myotube, samples (32). FSHD LCLs had high expression of both early and late DUX4 target genes in a manner that correlates with *DUX4* expression. However, FSHD myoblasts only expressed late DUX4 target genes, implying historic expression of *DUX4*. FSHD myotubes expressed both early and late DUX4 target genes, but in a manner uncorrelated with *DUX4* expression, so consistent with a transient *DUX4* pulse during differentiation. We also derived an FSHD lymphoblast biomarker of 237 up-regulated genes in FSHD LCLs, which is unaltered between FSHD and control myoblasts or myotube samples, showing that it is not associated with myogenic FSHD cells. There was significant up-regulation of our FSHD Lymphoblast score by meta-analysis over transcriptomic studies of seven independent FSHD muscle biopsy datasets, which was significantly correlated with expression of DUX4 target genes. Our FSHD lymphoblast biomarker also associated specifically with microscopic histological inflammation, while late DUX4 target gene expression associated with macroscopic MRI-based, STIR-positive inflammation.

In summary, *DUX4*-expressing lymphoblasts contribute significantly to the gene expression profile of FSHD muscle biopsies, being associated with early inflammatory changes, at a time when therapeutic intervention may prevent irreversible change.

## Results

### FSHD LCLs display robust DUX4 expression

**Figure 1:**
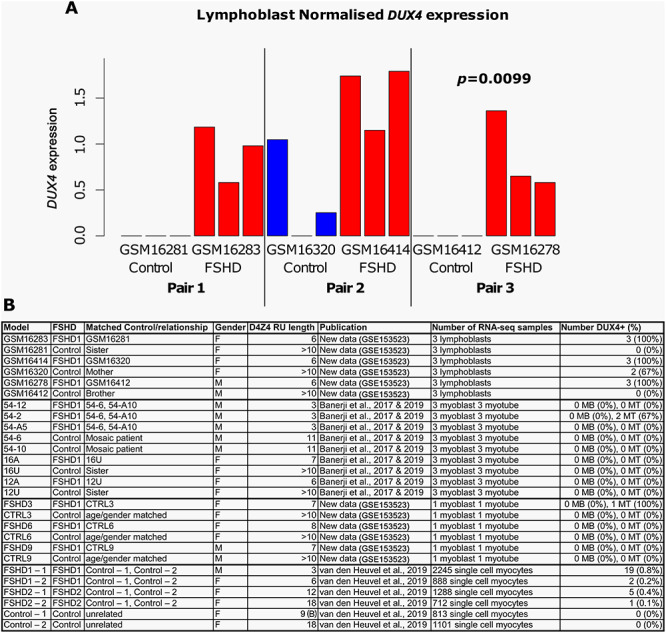
*DUX4* expression is robustly detected in RNA-seq of FSHD LCLs. (**A**) A bar plot displays normalized *DUX4* expression in our RNA-seq of three FSHD LCLs and first-degree relative matched controls for each sample profiled in triplicate. The *P*-value denotes the significance of differential expression analysis performed using the DESeq2 package in R, after adjustment for sex and matched pair. (**B**) A table summarizes *DUX4* expression in RNA-seq data corresponding to FSHD cellular models. Myoblast and differentiated myotube data are either new data (primary cell lines - GSE153523) or data previously published by ourselves in Banerji *et al.*, 2017 and 2019 (32, 45). Single cell RNA-seq of FSHD and control myocytes were previously published by van den Heuvel *et al*., 2019 (40). A sample was assessed as DUX4 positive if a single DUX4 read was found in normalized RNA-seq data.

From the LCL cohort generated by Jacobsen *et al*. (41) and further characterized by Jones *et al*. (42), we selected three clinically and genetically diagnosed FSHD1 patients with the expected degree of D4Z4 demethylation and robust *DUX4* expression. Controls were sex-matched first-degree relatives that had `healthy' levels of D4Z4 methylation and negligible *DUX4* expression. FSHD1 GSM16283 (6 repeat units (RU), female, family 2) with matched control GSM16281 (sister); FSHD1 GSM16278 (6RU, male, family 2) with matched control GSM16412 (brother) and the related FSHD1 GSM16414 (6RU, female, family 11) with matched control GSM16320 (mother) (42). RNA-seq was performed on each cell line in triplicate. *DUX4* transcripts were detected by RNA-seq in all FSHD LCL samples (9/9, 100%). *DUX4* transcripts were also present in 2/3 replicates of control LCL GSM16320 (2/9, 22%), although at significantly lower levels than its matched FSHD LCL GSM16414 (Fig. 1A). After adjusting for sex and patient control pair, we found that *DUX4* expression was significantly higher in FSHD LCLs compared to controls (*P =* 0.0099).

We also performed RNA-seq in singlet on three primary FSHD myoblast cell lines described previously (24), namely FSHD3 (FSHD1, 7RU, female), FSHD6 (FSHD1, 8RU, female) and FSHD9 (FSHD1, 7RU, male) alongside age and sex-matched controls, both in proliferation and after 3 days of differentiation into multinucleated myotubes. These new RNA-seq data were considered with our previously published datasets of immortalized FSHD myoblasts and myotubes in triplicate (32,45) that describes three pathological FSHD cell lines (54-12, 54-A5 and 54-2, all FSHD1, 3RU, male) alongside two control lines (54-A10, 54-6, 11RU) from a mosaic patient (46) and two further FSHD cell lines (16Abic, FSHD1, 7RU, female and 12Abic, FSHD1, 6RU, female) alongside sibling and sex-matched controls (16Ubic and 12Ubic, respectively). This totals 27 immortalized myoblasts and 27 immortalized myotube RNA-seq samples. None of the primary or immortalized FSHD myoblast or control myoblast samples contained *DUX4* transcripts detectable by RNA-seq (Fig. 1B). Considering the myotube transcriptomes, three FSHD myotube samples contained *DUX4* transcripts, namely primary line FSHD3 and 2/3 replicates of the immortalized 54-2 FSHD cell line (Fig. 1B). No control myotube samples expressed *DUX4* (Fig. 1B). In a recent single cell RNA-seq of FSHD1 and FSHD2 unfused myocytes, *DUX4* transcripts were found in 27/5133 (0.5%) FSHD cells (40) (Fig. 1B).

### FSHD LCLs and FSHD myotubes express early and late DUX4 target genes while FSHD myoblasts express only late DUX4 target genes

We next considered expression of DUX4 target genes in our LCL, myoblast and myotube transcriptomic data. We previously described three DUX4 target gene expression signatures derived from transcriptomic analysis of human myoblasts over-expressing DUX4 for different lengths of time (32). A set of 212 DUX4 target genes were derived from data described by Choi *et al*. (47) in which DUX4 was induced in a genetically modified control myoblast line for 8 h before samples were collected in triplicate for RNA-seq alongside uninduced controls. Thus, the Choi *et al*. DUX4 target gene expression signature represents early DUX4 target genes (47).

Another set of 165 DUX4 target genes was derived from data described by Geng *et al*. (29), in which control myoblasts were transduced by either a *DUX4*-encoding, or control, lentiviral vector and samples collected in quadruplicate 24 h later for microarray analysis. Thus, the Geng *et al*. DUX4 target gene expression signature represents later DUX4 target genes. A further 114 DUX4 target gene signature was described by Yao *et al*. (30). RNA-seq data used to derive this signature correspond to two different myoblast lines: 54-1 transfected with a *DUX4*-encoding lentivirus for 48 h and MB135 transfected with *DUX4*-encoding lentivirus for 24 h, alongside 54-1 untransfected control (though with reads from a *DUX4* expressing sample) and MB135 transfected with GFP lentivirus for 24 h (48). We consider the Geng *et al*. (24 h) and Yao *et al*. (24–48 h) as late DUX4 target gene signatures.

**Figure 2:**
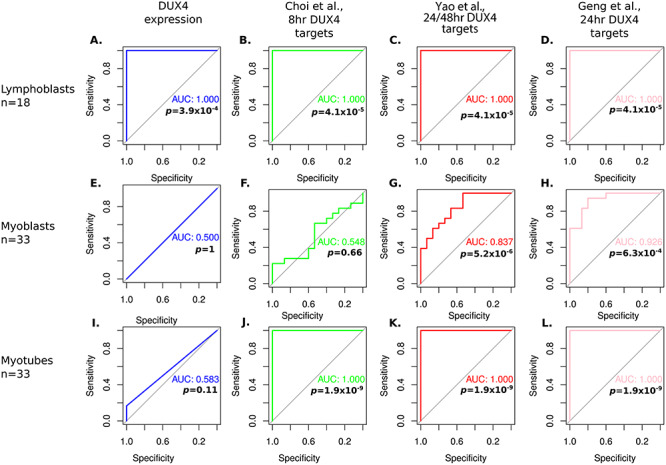
*DUX4* and early and late DUX4 target gene expression identifies FSHD LCLs more robustly than FSHD myoblasts or myotubes. (**A**–**L**) ROC curves display the discriminatory power of *DUX4* expression or expression of DUX4 target genes in patient derived LCLs (A–D), myoblasts (E–H) or differentiated myotubes (I–L), using the early Choi *et al*. (8 h) DUX4 target gene signature, or the late Yao *et al*. (24–48 h) and Geng *et al*. (24 h) DUX4 target gene signatures (all *z-*normalized within FSHD patient-matched control group within cell type). Only on LCLs are all four biomarkers perfect discriminators of FSHD status. AUC for each discriminator in each cell line is displayed alongside Wilcoxon *P*-values comparing the normalized biomarker value in FSHD samples vs controls.

For *DUX4* and each of the three DUX4 target gene expression signatures, we computed the mean expression of the genes in each LCL, myoblast or myotube sample, to generate a single sample score, as previously described (32,33). Scores were then *z*-normalized within patient-matched control groups and their performances as biomarkers of FSHD status evaluated using receiver operating characteristic (ROC) curve analysis, which depicts performance of a binary classifier at different threshold values. True-positive rate (sensitivity) was plotted against the false-positive rate (1-specificity) at different threshold values to generate the ROC curve. Area under the curve (AUC) represents the probability that *DUX4*, and each of the three DUX4 target gene expression signatures, will on average discriminate FSHD LCLs, myoblasts or myotubes from their relative controls (Fig. 2).

*DUX4* expression and each of the three DUX4 target gene expression signatures derived from ectopic *DUX4* expression in myogenic cells were perfect classifiers of FSHD status in LCLs (FSHD vs control: Wilcoxon *P* < 3.9 × 10^−4^, AUC = 1, *n* = 18 (9 FSHD, 9 controls), Fig. 2A–D). For myoblasts, no sample expressed *DUX4*, and the Choi *et al*. early (8 h) DUX4 target gene expression signature was not a significant classifier of FSHD status (FSHD vs control: Wilcoxon *P* = 0.66, AUC = 0.548, *n* = 33 (18 FSHD, 15 control), Fig. 2E and F). However, both the late Geng *et al*. (24 h) and Yao *et al*. (24–48 h), DUX4 target gene signatures were significant classifiers of FSHD myoblasts (Yao *et al*. FSHD vs control: Wilcoxon *P* = 5.2 × 10^−6^, AUC = 0.837; Geng *et al*. FSHD vs control: Wilcoxon *P* = 6.3 × 10^−4^, AUC = 0.926, Fig. 2G, H). Therefore, although FSHD myoblasts do not express *DUX4*, nor have hallmarks of recent DUX4 target gene expression, they do express late DUX4 target genes, implying historic *DUX4* expression. For myotubes, *DUX4* expression did not represent a significant classifier of FSHD status (FSHD vs control: Wilcoxon *P* = 0.11, AUC = 0.583, *n* = 33 (18 FSHD, 15 control), Fig. 2I). However, both the early and the two late DUX4 target gene expression signatures were perfect classifiers of FSHD myotubes (FSHD vs control: all Wilcoxon *P* = 1.9 × 10^−9^, AUC = 1, Fig. 2J–L). This suggests that during myogenic differentiation, FSHD myoblasts express a transient pulse of *DUX4*, leading to activation of both early and late DUX4 targets by the end of differentiation, although DUX4 itself is no longer detectable at this stage.

DUX4 target genes that overlap between the Choi *et al*. early (8 h), and Geng *et al*./Yao *et al*. late (24–48 h) DUX4 signatures were also removed to determine if this increased the power of discrimination between FSHD and control (Supplementary Material, Figure S1). Early DUX4 target genes were defined as those exclusively in the Choi *et al*. DUX4 target gene set, but absent from both Yao *et al*. and Geng *et al*. DUX4 target gene sets. Early and late DUX4 target genes are those present in both Choi *et al*. and either Yao *et al*. or Geng *et al*. DUX4 target gene sets. Late DUX4 target genes are absent from the Choi *et al*. DUX4 target gene set, but present in either the Yao *et al*. or Geng *et al*. DUX4 target gene set. Removal of such overlapping DUX4 target genes did not dramaticaly change the power of discrimination between FSHD and control for each cell type. In general, the discriminatory power was similar to that using the full DUX4 target gene sets including overlaps, (Supplementary Material, Figure S1). As the full overlapping DUX4 target gene signatures more accurately describe genes induced by DUX4 at early and late time points, the full Choi *et al*. (8 h), Geng *et al*. (24 h) and Yao *et al*. (24–48 h) gene sets were used henceforth.

### DUX4 expression is correlated with early and late DUX4 target gene expression in FSHD LCLs but not in FSHD myotubes

**Figure 3:**
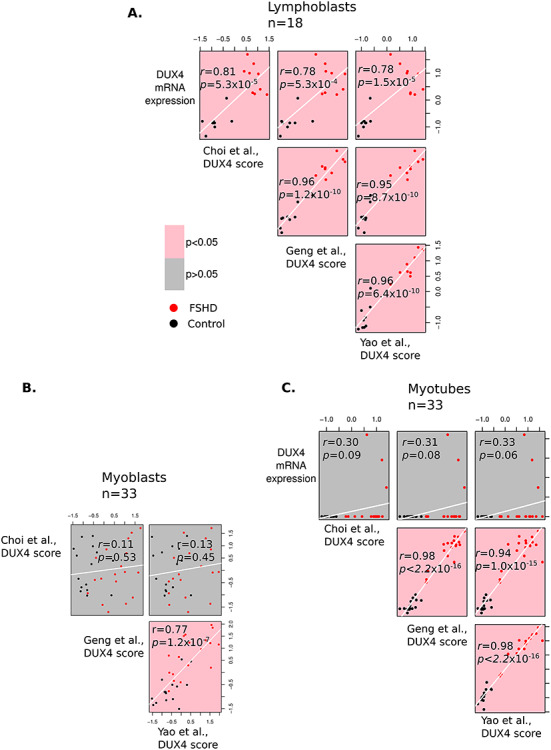
*DUX4* expression correlates with expression of early and late DUX4 target genes in LCLs but not in myoblasts or myotubes. (**A**–**C**) Scatter plots display *DUX4* expression and the early Choi *et al*. (8 h), and the late Yao *et al*. (24–48 h) and Geng *et al*. (24 h) DUX4 target gene signatures (all *z-*normalized within FSHD patient-matched control group within cell type) plotted against one another across the 18 LCL samples (A), the 33 myoblast samples (B) and the 33 myotube samples (C). Only on LCLs, all four DUX4 biomarkers are significantly correlated. Pearson’s *r* and associated *P*-value are provided for each pairwise comparison. Red points correspond to FSHD samples, while black points represent controls. Plots denoting correlations reaching significance are pink, whilst those not attaining significance are grey. Since no myoblast samples expressed *DUX4*, the *DUX4* mRNA expression comparison row is not displayed (B).

We next investigated how *DUX4* and DUX4 target genes correlated with one another within the different cell types. For LCLs, *DUX4* expression correlated strongly with both early and late DUX4 target gene expression (*DUX4* expression vs Choi *et al*. *P* = 5.3 × 10^−5^, Pearson’s *r =* 0.81, *DUX4* expression vs Geng *et al*. *P* = 5.3 × 10^−4^, Pearson’s *r =* 0.78, *DUX4* expression vs Yao *et al*. *P* = 1.5 × 10^−5^, Pearson’s *r =* 0.78, Fig. 3A). The early and late DUX4 target gene expression scores also correlated strongly in LCLs (Choi *et al*. vs Geng *et al*. *P* = 1.2 × 10^−10^, Pearson’s *r =* 0.96, Choi *et al*. vs Yao *et al*. *P* = 8.7 × 10^−10^, Pearson’s *r =* 0.95, Fig. 3A). This confirms that DUX4 target genes identified via exogenous *DUX4* expression in myoblasts associates with endogenous *DUX4* expression in FSHD LCLs, implying many common DUX4 target genes between the two cell types. This also suggests that some DUX4 target genes detected in FSHD muscle biopsies may be derived from infiltrated immune cells, as well as from muscle cells.

Myoblasts all lacked *DUX4* expression. The two late DUX4 target gene signatures of Geng *et al*. and Yao *et al*. correlated (Geng *et al*. vs Yao *et al*. *P* = 1.2 × 10^−7^, Pearson’s *r =* 0.77, Fig. 3B), confirming their reproducibility. However, the Choi *et al*. early DUX4 target gene signature was unrelated to these later DUX4 target gene sets (Choi *et al*. vs Geng *et al*. *P* = 0.53, Pearson’s *r =* 0.11, Choi *et al*. vs Yao *et al*. *P* = 0.45, Pearson’s *r =* 0.13, Fig. 3B). This indicates that *DUX4* expression in FSHD myoblasts was sufficiently historic that early DUX4 target gene expression is not related to persistent late DUX4 target gene activation.

There was no association between *DUX4* expression and any of the DUX4 target gene scores in myotubes (*DUX4* expression vs Choi *et al*. *P* = 0.09, Pearson’s *r =* 0.30, *DUX4* expression vs Geng *et al*. *P* = 0.08, Pearson’s *r =* 0.31, *DUX4* expression vs Yao *et al*. *P* = 0.06, Pearson’s *r =* 0.33 Fig. 3C), but this analysis is underpowered as only three myotube samples expressed *DUX4* (Fig. 1B). In contrast to myoblasts, however, there was a strong correlation between the early and late DUX4 target gene scores (Choi *et al*. vs Geng *et al*. *P <* 2.2 × 10^−16^, Pearson’s *r* = 0.98, Choi *et al*. vs Yao *et al*. *P* = 1.0 × 10^−15^, Pearson’s *r =* 0.94, Fig. 3C). This is consistent with a transient burst of *DUX4* expression during myogenic differentiation. FSHD myotube samples express significantly higher levels of both early and late DUX4 target genes than their corresponding FSHD myoblast samples (Wilcoxon *P ≤* 2.5 × 10^−4^Supplementary Material, Figure S2A–C). Control myotubes displayed significantly lower levels of the early DUX4 target genes to their corresponding myoblast samples (Wilcoxon *P =* 0.002, Supplementary Material, Figure S2D), but similar levels of late DUX4 target genes (Wilcoxon *P* ≥ 0.3, Supplementary Material, Figure S2E, F).

We previously evaluated the discriminatory power of the three DUX4 target gene scores on unfused FSHD myocytes profiled by single cell RNA-seq, and although significant discriminators, no score achieved an AUC > 0.56 (33). However, 27/5133 myocytes from the four FSHD patients expressed *DUX4* (40). This offers greater power for assessment of *DUX4* association with the DUX4 target gene scores in differentiated muscle cells, than just using the three myotube samples with DUX4 transcripts (Fig. 3C**)**. The early and late DUX4 target gene expression scores correlated in this single cell dataset (Choi *et al*. vs Geng *et al*. *P <* 2.2 × 10^−16^, Pearson’s *r* = 0.54, Choi *et al*. vs Yao *et al*. *P* < 2.2 × 10^−16^, Pearson’s *r =* 0.38, Geng *et al*. vs Yao *et al*. *P* < 2.2 × 10^−16^, Pearson’s *r =* 0.86 Supplementary Material, Figure S3). Surprisingly though, *DUX4* expression was again not associated with either early or late DUX4 targets in single FSHD myocytes (*DUX4* expression vs Choi *et al*. *P* = 0.8, Pearson’s *r =* 0.16, *DUX4* expression vs Geng *et al*. *P* = 0.6, Pearson’s *r =* 0.22, *DUX4* expression vs Yao *et al*. *P* = 0.5, Pearson’s *r =* 0.23, Supplementary Material, Figure S3). Plotting *DUX4* expression against early and late DUX4 target gene scores in the single cell data reveals a peak of *DUX4* expression in cells with low levels of DUX4 target genes. However, *DUX4* expression then decays as DUX4 target genes increase (Supplementary Material, Figure S3). This is consistent with a transient pulse of *DUX4* expression in differentiating FSHD myoblasts, which shuts down as DUX4 target genes are activated.

### An FSHD lymphoblast signature is up-regulated in FSHD muscle biopsies and correlates with DUX4 target gene expression

Given that FSHD LCLs have expression of *DUX4* and both early and late DUX4 target genes, and that FSHD muscle biopsies are often characterized by inflammation in a manner correlating with DUX4 target gene expression (33,49), we next investigated whether an FSHD LCL-derived gene expression signature can discriminate FSHD muscle biopsies from controls.

We performed a differential expression analysis comparing FSHD LCLs to controls, adjusting for sex and sibling matched pairs. The FSHD and control lymphoblastoid cell lines are all EBV-immortalized, and so genes associated with immortalization are common to both and should not feature in our LCL signature. We identified a large number of differentially expressed genes and considered the 500 most significantly altered for further analysis. Of these, 237/500 were up-regulated in FSHD LCLs. DUX4 is a known transcriptional activator and genes suppressed under *DUX4* expression in myoblasts does not add power to DUX4 target gene-based FSHD biomarkers (32). Therefore, we considered mean expression of these 237 FSHD LCL up-regulated genes to generate the FSHD Lymphoblast score (Supplementary Material, Table S1). Of these 237 genes, 9 were also present in Choi *et al*. and 1 in the Geng *et al*. DUX4 target gene signatures, but none in Yao *et al*. The full FSHD Lymphoblast score is used here, since results were unchanged when these DUX4 target genes were removed.

A Gene Set Enrichment Analysis (GSEA) for genes of the FSHD Lymphoblast score showed pathways enriched were related to B-cell differentiation, T-reg cells and viral/vaccine response (Supplementary Material, Table S2**)**. Our lymphoblastoid-specific FSHD score was also enriched for genes up-regulated in stem cells and involved in EZH2 misregulation (Supplementary Material, Table S2**)**, in line with our previous investigations into DUX4 function (26) and FSHD muscle biopsy gene expression (32).

**Figure 4:**
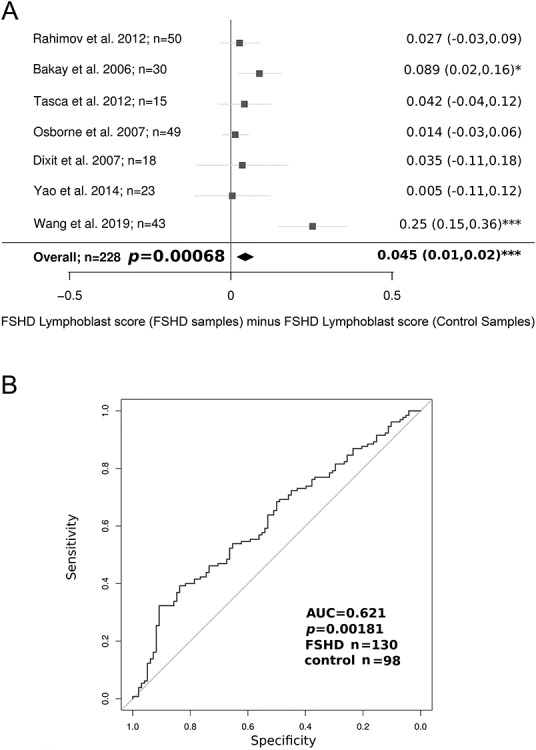
The FSHD Lymphoblast score is elevated on FSHD muscle biopsies compared to controls on meta-analysis of seven independent datasets. (**A**) Forest plot displays the significance of the FSHD Lymphoblast score as a discriminator of FSHD muscle biopsies in seven independent microarray or RNA-seq datasets (130 FSHD, 98 control). On meta-analysis, the FSHD Lymphoblast score is elevated on FSHD samples. The FSHD Lymphoblast score achieves strongest significance on the Wang *et al*. (49) RNA-seq dataset, where areas of muscles displaying evidence of active disease on MRI were preferentially biopsied. Boxes denote the mean difference in FSHD Lymphoblast score between FSHD and control muscle biopsies and whiskers denote 95% confidence interval. A vertical line denotes a score difference of 0 and datasets where the whiskers cross this line have not attained significance at *P* < 0.05 (as assessed by Wilcoxon *U*-test). Numerical values for mean score difference and confidence interval are displayed for each dataset to the right of the plot with significance denoted by asterisks where ^*^ denotes *P* < 0.05, ^*^^*^ denotes *P* < 0.01 and ^*^^*^^*^ denotes *P* < 0.001. The overall estimate is displayed as a diamond and was computed using a random effects model with significance assessed via Fisher’s combined test. (**B**) A ROC curve displays the discriminatory capacity of the FSHD Lymphoblast score on all muscle biopsy datasets combined. The FSHD Lymphoblast score was computed on each muscle biopsy sample and *z*-normalized within each of the seven independent studies before being pooled for ROC curve analysis. The AUC of the FSHD Lymphoblast score as a discriminator of FSHD muscle biopsies is displayed alongside the Wilcoxon *P*-value comparing normalized FSHD Lymphoblast score values in FSHD muscle biopsies to controls.

**Figure 5:**
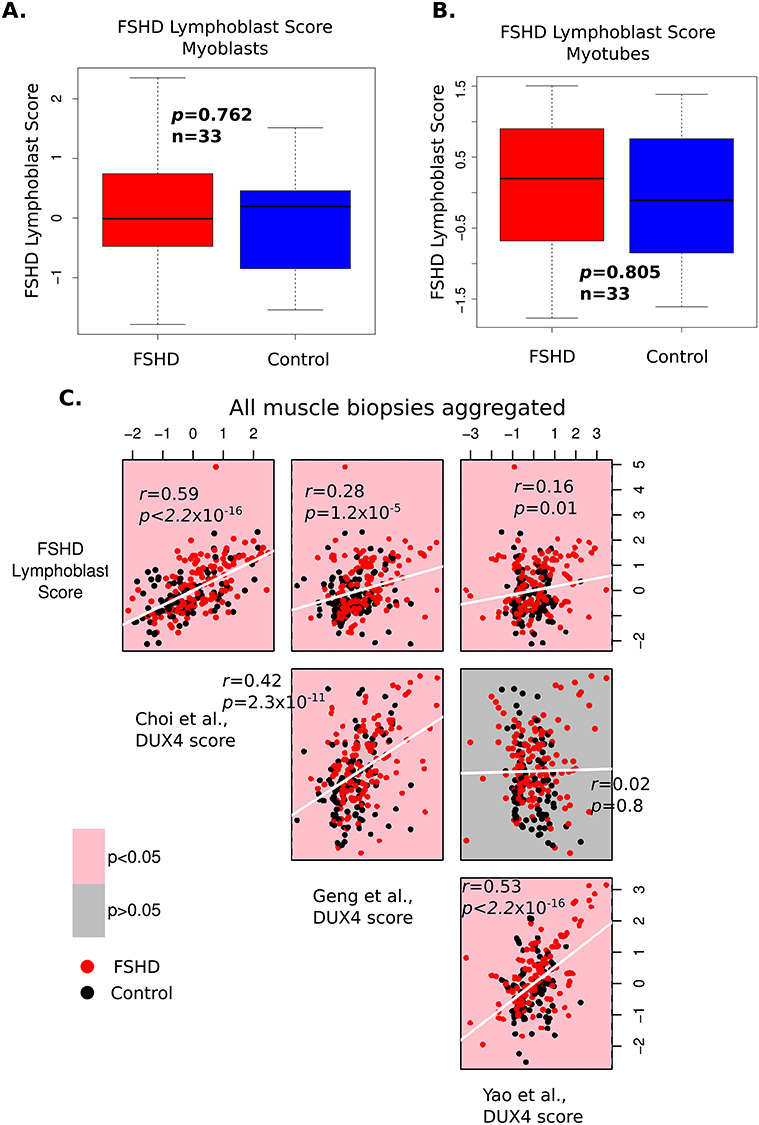
The FSHD Lymphoblast correlates with the level of DUX4 target gene expression in FSHD muscle biopsies. (**A** and **B**) Box plots display the FSHD Lymphoblast score (*z-*normalized within FSHD patient-matched control group within cell type) in FSHD and control myoblast samples (A) and myotube samples (B). The FSHD Lymphoblast score is not significantly altered in FSHD on either myoblasts or myotubes. The box represents the interquartile range (IQR), with the median indicated by a line. Whiskers denote min [1.5^*^IQR, max (observed value)]. Wilcoxon *U*-test *P*-values comparing FSHD to control samples are presented. (**C**) Scatter plots display the FSHD Lymphoblast score, the early Choi *et al*. (8 h), and late Yao *et al*. (24–48 h) and Geng *et al*. (24 h) DUX4 target gene signatures (all *z-*normalized within each of the seven muscle biopsy studies) plotted against one another across all 228 muscle biopsies (130 FSHD, 98 control). The FSHD Lymphoblast score correlates with all the DUX4 target gene expression scores but most strongly with the early DUX4 target gene signature of Choi *et al*. Pearson’s *r* and associated *P*-value is provided for each pairwise comparison. Red points correspond to FSHD samples, while black points represent controls. Plots denoting correlations reaching significance are pink, whilst those not attaining significance are grey.

The FSHD Lymphoblast score was evaluated on each sample of seven independent FSHD muscle biopsy transcriptomic studies (22,30,49–53), totalling 130 FSHD samples alongside 98 matched controls. The FSHD Lymphoblast score was significantly up-regulated in FSHD muscle biopsies on meta-analysis (Fisher’s combined *P* = 0.0007, Fig. 4A), achieving outright significance on two datasets, and representing a moderately powered biomarker of FSHD status under ROC curve analysis (Wilcoxon *P* = 0.0018, AUC = 0.621, Fig. 4B). Of the FSHD muscle biopsy datasets, the strongest up-regulation of the FSHD Lymphoblast score was found in the MRI-guided RNA-seq dataset (49), in which all but two FSHD samples displayed STIR positivity, indicative of active inflammation [Wang *et al*. (49), Wilcoxon *P* < 1.5 × 10^−5^, Fig. 4A]. Importantly, up-regulation of the FSHD Lymphoblast score in FSHD muscle biopsies is unlikely to be driven by muscle gene expression, since there was no significant difference in expression of the FSHD Lymphoblast score on our RNA-seq data of FSHD and control myoblasts (Wilcoxon *P* = 0.76, Fig. 5A) or myotubes (Wilcoxon *P* = 0.81, Fig. 5B).

ROC curve analysis shows that the three DUX4 target gene scores are weak, but significant discriminators of FSHD status, statistically equivalent to the FSHD Lymphoblast score, but all are inferior classifiers of FSHD muscle biopsies to PAX7 target gene repression (32,33) using DeLong’s test (Supplementary Material, Figure S4). Evaluating associations between the FSHD Lymphoblast score and the three DUX4 target gene expression scores across the FSHD muscle biopsies revealed that the FSHD Lymphoblast score strongly associated with the Choi *et al*. early DUX4 target genes (FSHD Lymphoblast score vs Choi *et al*. *P* < 2.2 × 10^−16^, Pearson’s *r =* 0.59, Fig. 5C). A weaker but significant association was found between the FSHD Lymphoblast score and the two late DUX4 target gene expression signatures (FSHD Lymphoblast score vs Geng *et al*. *P* = 1.2 × 10^−5^, Pearson’s *r =* 0.28, FSHD Lymphoblast score vs Yao *et al*. *P* = 0.01, Pearson’s *r =* 0.16 Fig. 5C).

### The FSHD Lymphoblast score is associated with histological inflammation in FSHD muscle biopsies, independently of DUX4 target gene expression

**Figure 6:**
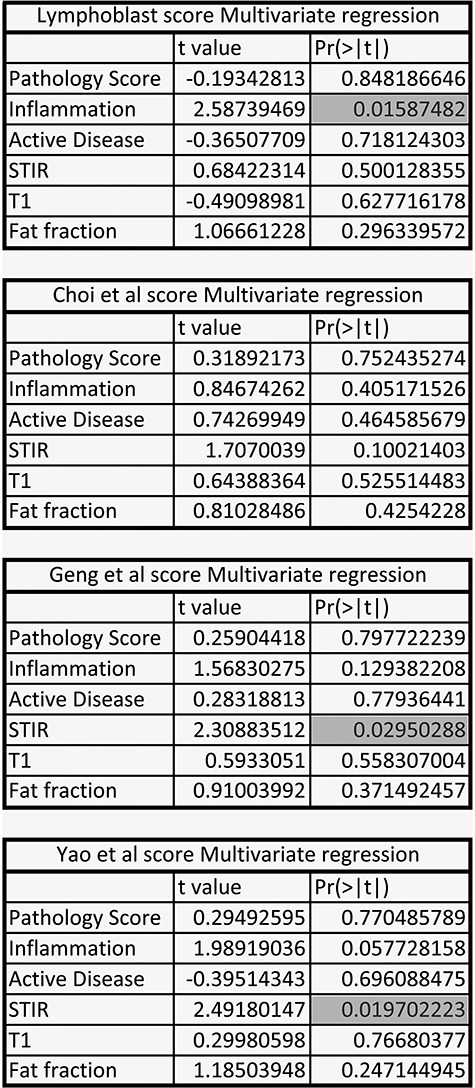
The FSHD Lymphoblast score correlates specifically with histological inflammation in FSHD patient muscle biopsies, while the late DUX4 target gene expression scores correlate with STIR positivity on MRI. Tables summarize multivariate regression analyses of the FSHD Lymphoblast score and each of the three DUX4 target gene signatures on the dataset described by Wang *et al*. (49) determining the independent association of histological (Pathology score, Inflammation, Active Disease) and MRI-based (STIR, T1, fat fraction) assessments of FSHD disease activity. The FSHD Lymphoblast score is only independently associated with histological inflammation. The Choi *et al*. early DUX4 target signature is not independently associated with any measure of disease activity. The two late DUX4 target gene signatures (Yao *et al*. and Geng *et al*.) both associate with the level of STIR positivity. Multivariate regression *t*-values and associated *P*-values are provided for each of the FSHD disease activity variables association with each score separately, *P*-values attaining significance at *P* < 0.05 are highlighted.

FSHD LCL gene expression is elevated in FSHD muscle biopsies (Fig. 4) but not in FSHD myoblasts or myotubes (Fig. 5A and B), suggesting that the FSHD Lymphoblast score may be detecting immune cell infiltrates in FSHD muscle biopsies. To investigate, we considered published RNA-seq data of FSHD muscle biopsies alongside histological assessment of pathology score, inflammation and active disease, together with MRI assessment of STIR and T1 positivity and fat fraction (49). Histological and MRI assessments are all metrics of active pathology in FSHD and hence cross-correlate. We therefore built multivariate regression models evaluating which of these variables were independently associated with the FSHD Lymphoblast score, or each of the three DUX4 target-gene expression signatures (Fig. 6**)**.

Crucially, the FSHD Lymphoblast score associated only with histological inflammation (*P* = 0.016, Fig. 6), indicating that our score does indeed correlate with immune cell infiltration of FSHD muscle biopsies. Early DUX4 target genes (Choi *et al*. 8 h) did not independently associate with any of the measures of active pathology in FSHD. However, the two late DUX4 target gene expression signatures (24–48 h) both significantly associated with STIR positivity (Geng *et al*. *P* = 0.030, Yao *et al*. *P* = 0.020, Fig. 6).

## Discussion

FSHD is an enigmatic pathology, characterized by considerable heterogeneity and complex molecular pathophysiology (14). Despite this, consensus has emerged on the causal role of DUX4 in driving FSHD pathology: a theory underpinned by the epigenetic derepression at D4Z4 that characterizes both FSHD1 and FSHD2 (13,18,19). However, understanding how DUX4 causes pathology has proven difficult. FSHD presents as a skeletal muscular dystrophy, hence studies into the function of DUX4 in FSHD have typically focused on myogenic cells (27,29–31,47). *DUX4* is very difficult to detect in FSHD muscle tissue though, generally requiring techniques such as nested RT-qPCR, with immunolabelling detecting DUX4 in as few as 1/1000 proliferating FSHD myoblasts ex vivo (31,33). Indeed, we were unable to detect *DUX4* transcripts in any of 18 FSHD immortalized or primary myoblast samples by RNA-seq, and in only 3/18 myotube samples.

While investigation of FSHD myogenic cells is important, muscle is not a homogenous tissue. Given that epigenetic derepression at D4Z4 and *DUX4* expression occurs in other cell types in FSHD (31,54), it is possible that non-myogenic cells also express *DUX4* in muscle tissue. Pathological skeletal muscle damage observed in FSHD may not solely be driven by DUX4 in myofibres, but also by aberrant inflammation and vascularization of muscle. FSHD muscle biopsies are characterized by lymphocytic infiltrates, particularly of endomysial (CD8^+^) and perivascular (CD4^+^) T lymphocytes (35), while capillary density is significantly lower (55). Most of the FSHD patient-derived LCL cohorts from 12 multigenerational FSHD families (41) express significant, but variable, levels of endogenous *DUX4-fl*, with a good correlation between DNA hypomethylation and D4Z4 repeat length (42).

We found that the six LCL lines and matched controls that we selected had high expression of *CD20* but low/negligible expression of *CD3*, *CD4* or *CD8* via our RNA-Seq, consistent with a B-cell classification. FSHD patient-derived LCLs express endogenous *DUX4*, together with early and late DUX4 target genes (identified from exogenous DUX4 expression in myogenic cells) more robustly than FSHD myoblasts or differentiated myotubes. We also identified 237 genes that are up-regulated in FSHD LCLs compared to controls that we termed the FSHD Lymphoblast score. Since both the FSHD and control lymphoblastoid cells lines were EBV immortalized, genes associated with immortalization (56) are common to both, so should not feature in our FSHD Lymphoblast score. Immortalized LCLs often recapitulate the profile of native gene expression in primary B cells, with only small variance detected in most gene expression levels between LCLs and primary B cells (57). EBV immortalization, however, maintains proliferating LCLs with transcriptomic (up-regulation of *IRF4*, *PRDM1/BLIMP1* and *XBP1,* but maintenance of *CD20/MS4A1* and *PAX5*) and phenotypic features that are similar to plasmablasts and early plasma cells, which are normally only transient stages in B cell differentiation (56), hence the ‘lymphoblastoid’ designation. Despite these caveats, the mean expression of the genes of the FSHD Lymphoblast score is elevated in FSHD muscle biopsies, where they associate strongly with histological assessment of inflammatory infiltrates of primary immune cells. This is supported by the observation that the FSHD Lymphoblast score is unaltered between FSHD and control immortalized/primary myoblasts or myotubes. Interestingly, the FSHD Lymphoblast score correlates with both early and late DUX4 target gene activation in FSHD muscle biopsies, with 10 of the 237 genes being DUX4 target genes identified from muscle cells. Since FSHD lymphoblastoid cells express *DUX4* and some DUX4 target genes at constitutive high levels, the distinctive lymphocytic infiltration in FSHD muscle biopsies may contribute to DUX4 target gene expression. It would be highly informative to analyze *DUX4*/DUX4 target genes directly in primary muscle-infiltrating immune cells.

The muscle cell contribution to *DUX4*/DUX4 target genes in FSHD muscle biopsies is probably via dynamic, stochastic *DUX4* expression (58) rather than the continuous expression measured in lymphoblastoids (42). Such transient bursts of *DUX4* expression presumably occur in mature muscle fibres to elicit myofibre damage, but *DUX4* could also be expressed during any subsequent regenerative response. The resident stem cell of skeletal muscle is the satellite cell (59). *DUX4* expression in satellite cells will have direct deleterious effects via transcriptional activation of DUX4 target genes that inhibit myogenic differentiation and promote apoptosis (24–27). DUX4 can also operate via interference with the normal function of PAX7 in myoblasts (32). By these pathomechanisms, DUX4 could compromise regenerative myogenesis and so the muscle repair response in FSHD (40,58,60). We show that FSHD myoblasts lack expression of both *DUX4* itself and early DUX4 target genes, but exhibit clear up-regulation of late DUX4 target genes: indicating a historic, transient expression of *DUX4*. A transient DUX4 expression profile in satellite cell-derived myoblasts could explain the robust repression of PAX7 target genes seen in FSHD muscle biopsies (32,33).

We further demonstrate that *DUX4* is detectible by RNA-seq in 17% (3/18) of FSHD myotube samples, with myotubes displaying distinct up-regulation of both early and late DUX4 target genes, compared to their corresponding myoblast samples. This is consistent with the reported pulse of *DUX4* expression and DUX4 target genes during myogenic differentiation (58,60,61), and further supported by a burst-like expression pattern of *DUX4* that we find when examining published RNA-seq of single FSHD unfused myocytes (40). Such dynamic *DUX4* up-regulation may contribute to the modest efficacy of DUX4 target gene expression as a biomarker in FSHD muscle biopsies (32), but this could be in combination with contributions from *DUX4* expressing immune cells.

Our findings have a number of implications. The first relates to DUX4 function and role in pathology. Currently, investigation of DUX4 target genes in FSHD has been performed in myoblast cell lines (26,29,30,47,62) where DUX4 and its target genes lead to pro-apoptotic and anti-myogenic effects (26–28,47). Interestingly, LCL lines proliferate in the presence of endogenous *DUX4* expression and both early and late DUX4 target genes, and so seem more refractory to the apoptosis normally induced by DUX4 in myogenic cells, and many other cell type cells (28,63). Moreover, differential white cell counts in FSHD patient peripheral blood shows no significant differences in absolute numbers of B-cells compared to controls, but a raised CD8^+^ cell count (35). Genes associated with the immune system are also dysregulated by DUX4 in myogenic cells (29), and DUX4 promotes immune evasion in cancer cells by blocking interferon-γ regulated major histocompatibility complex class 1 genes, so reducing antigen presentation (64). In addition, a DUX4-IGH fusion gene is present in a significant proportion of adult B-cell acute lymphoblastic leukaemia patients, where it binds DUX4 response elements and alters the canonical gene expression profile (43,44). Since *DUX4* is continuously expressed in FSHD LCLs and early and late DUX4 target genes are present, this implies modification of immune cell function. Suppressing DUX4 is currently the focus of several studies/trials into potential therapeutic strategies for FSHD (65–67) and so muscle-localized immune cells, as well as myogenic cells, may need to be targeted.

Histological and MRI analysis have long pointed to a role for inflammation in contributing to FSHD muscle damage (35,49,53). Our FSHD Lymphoblast score correlates with such inflammation, associating specifically with early, microscopic histological inflammation in FSHD muscle biopsies. In contrast, the two late DUX4 target gene cohorts of Geng *et al*. (29) and Yao *et al*. (30) both associate with later macroscopic inflammation, as assessed by STIR positivity on MRI. Thus, the FSHD Lymphoblast score may be a superior biomarker to late DUX4 target gene expression biomarkers in detection of the early stages of FSHD pathological inflammation, at a time when it is possibly reversible. Although anti-inflammatory agents such as corticosteroids have been used in clinical trials for FSHD without obvious benefit, the premise was that the inflammation was secondary to muscle pathology and effects on long-term disease progression were not assessed (68). Moreover, expression of *DUX4* and its target genes in muscle-infiltrated lymphocytes would change their global gene expression profile and alter cellular function, which could render them directly pathogenic. As such, if infiltrated lymphocytes are a primary driver of FSHD, rather than a secondary response, they may require more bespoke therapeutic interventions (69,70).

To summarize, we demonstrate that immortalized FSHD LCLs continuously express endogenous *DUX4*, together with early and late DUX4 target genes, in contrast to a burst-like *DUX4* expression pattern in myogenic cells. Our FSHD Lymphoblast score correlates with early stages of muscle inflammation, while our two late DUX4 target gene expression biomarkers associate with more pronounced inflammation. Therefore as DUX4-expressing immortalized FSHD lymphoblastoid cells express genes elevated in FSHD muscle biopsies, muscle-infiltrated immune cells likely contribute to the molecular landscape of FSHD.

## Materials and Methods

### Cell culture of FSHD LCLs and primary myoblasts

LCLs were originally derived from peripheral blood leucocytes isolated from clinically diagnosed FSHD patients and matched family controls via centrifugation (histopaque gradient) before transformation using Epstein–Barr virus (41). LCLs were subsequently genetically confirmed as being from FSHD patients and both the degree of demethylation at D4Z4 and relative DUX4 expression determined (42).

LCLs were obtained from NIGMS Human Genetic Cell Repository at the Coriell Institute for Medical Research (CIMR) repository, NJ 08103, USA. Lymphoblastoid FSHD cell lines GSM16283, GSM16414, GSM16278 and respective matched control lines GSM16281, GSM16320, GSM16412 were from two directly related families from Southern Utah, USA. LCLs were cultured in suspension in RPMI-1640 medium, supplemented with L-glutamine, sodium bicarbonate (Sigma), 10% fetal bovine serum (FBS) (Sigma) and gentamycin (Gibco). Cell pellets were collected from three independent flasks for each cell line.

Cell pellets corresponding to FSHD primary myoblast cell lines FSHD3 (FSHD1, 7RU, female), FSHD6 (FSHD1, 8RU, female) and FSHD9 (FSHD1, 7RU, male) alongside age and sex matched controls (24), in proliferation and after 3 days of differentiation into multinucleated myotubes, in singlet, were kind gifts from Dr Dalila Laoudj-Chenivesse (University of Montpellier, Montpellier, France).

### RNA-sequencing of FSHD LCLs and primary myoblasts

RNA was isolated using miRNAeasy kit (Qiagen) including a DNAse digestion step. RNA was analyzed by LabChip Bioanalyzer, Qubit fluorometric quantification and Nanodrop quantification of concentration and stability. RNA-seq libraries were prepared using the sureselect-stranded RNAseq protocol (Illumina), which allows polyA selection but was modified to work with ribodepletion (Agilent). Libraries were sequenced on an Illumina HiSeq2500.

Raw reads were trimmed using trim-galore, utilizing cutadapt14 (v0.4.0) to remove the Illumina Sequencing Adapter (AGATCGGAAGAGC) at the 3′ end. Additionally, 12 bases were also trimmed from the 5′ end, in both myoblast and LCL samples and five bases from the 3′ end in the LCL samples, since they showed a biased distribution. Reads were mapped to the human transcriptome using the human genome sequence GRCh38 and v82 gene annotations downloaded from Ensembl. Mapping was performed using tophat 15 (v2.1.0) and bowtie 16 (v1.1.0), enabling the fr-firststrand option of tophat to restrict mapping to the sense strand of the transcript. Reads were assigned to genes using the featureCounts program 17 (v1.5.0), counting fragments and ignoring multi-mapping reads, and restricted to the sense strand. The resulting matrix of read counts was analyzed using R.

Data describing the myoblast and LCLs were processed in separate batches and therefore analyzed as separate datasets. Both datasets were normalized using the DESeq2 package (71) in R. New RNA-Seq data is available at GSE153523.

### Public data on FSHD myoblasts, myotubes and muscle biopsies

Data containing myoblast and myotube RNA-seq samples in triplicate from immortalized FSHD myoblast cell lines 54-2, 54-12, 54-A5, 16ABic and 16UBic and matched controls 54-A10, 54-6, 16UBic and 12UBic that we previously described (32,45) are available from the GEO database, accession numbers: GSE123468 and GSE102812. These data describe 27 (15 FSHD, 12 control) myoblast samples and 27 (15 FSHD, 12 control) myotube samples.

Data containing RNA-seq of 7234 (5133 FSHD, 2101 control) single myocytes were described by van den Heuvel *et al*. (40), and normalized read counts were downloaded from GEO database accession GSE122873.

Seven datasets containing transcriptomic assessments of muscle biopsies were analyzed, and all were downloaded as normalized datasets from the GEO database. Rahimov *et al*. (50), GSE36398, describe 50 muscle biopsies assessed by microarray. Bakay *et al*. (52), GSE3307, describe 30 muscle biopsies assessed by microarray. Tasca *et al*. (53), GSE26852, describe 15 muscle biopsies assessed by microarray. Osborne *et al*. (51), GSE10760, describe 49 muscle biopsies assessed by microarray. Dixit *et al*. (22), GSE9397, describe 18 muscle biopsies assessed by microarray. Yao *et al*. (30), GSE56787, describe 23 muscle biopsies assessed by RNA-seq (control sample C6 was removed as it was the only non-quadriceps sample). Wang *et al*. (49), GSE115650, describe 43 muscle biopsies assessed by RNA-seq. Together, these seven datasets describe 228 muscle biopsies (130 FSHD, 98 control).

All data were log-transformed and quantile normalized within study for computation of the DUX4, FSHD Lymphoblast and PAX7 scores, in line with our previously described methodology (32).

### DUX4 detection, differential expression analysis and derivation of the FSHD Lymphoblast score

DUX4 detection was reported as positive if a single read was present in the normalized RNA-seq dataset. Differential expression analysis of the LCL data was performed using the DESeq2 package in R (71) to identify genes associated with FSHD independently of sex and matched-control pair, feature significance was confirmed via *P*-value histogram. The top 500 significant genes were considered for further analysis. The FSHD LCLs were found to express high levels of DUX4 and DUX4 target genes, and DUX4 is a transcriptional activator with repressed genes adding no power in previous FSHD biomarkers (32). We thus considered the mean expression of the 237/500 genes that were up-regulated in FSHD LCLs in a given sample, as a potential FSHD biomarker, referred to as the FSHD Lymphoblast score.

### Statistics: biomarker computation and evaluation

Computation of the three DUX4 expression biomarkers and PAX7 target gene repression biomarker were as previously described (32,33). Briefly, each DUX4 target gene expression score is computed for each sample as the mean expression of the genes found to be up-regulated by the studies of Yao *et al*. (30) (114 genes), Geng *et al*. (29) (165 genes) and Choi *et al*. (47) (212 genes). The PAX7 target gene repression score for each sample was computed as the *t*-score from a test comparing the up-regulated (311 genes) to down-regulated (290 genes) PAX7 target genes within each sample. We have published a software for the computation of each of these scores from suitably normalized dataset (33). The FSHD Lymphoblast score was computed in each sample as the mean expression of the 237 genes found up-regulated in FSHD LCLs.

For myoblast, myotube and LCL samples, the three DUX4 scores and the FSHD Lymphoblast score were evaluated and *z*-normalized within matched control pairs. Score differences between FSHD and controls samples were then evaluated within each cell type via a Wilcoxon *U*-test. ROC curve analysis and AUC computation were performed using the pROC package in R (72).

For FSHD muscle biopsy samples, the three DUX4 scores, the FSHD Lymphoblast score and the PAX7 score were computed for each sample and *z*-normalized within each of the seven studies. Score differences between FSHD and control samples were evaluated within each study via Wilcoxon *U*-test. In the case of the FSHD Lymphoblast score, meta-analysis across the seven independent studies were performed using a random effects model, and overall significance assessed via Fisher’s combined test. ROC curve analysis, AUC computation and DeLong’s test were performed using all *z*-normalized scores for all studies combined, via the pROC package in R (72).

### Statistics: correlation analyses

Pearson correlations between the three DUX4 scores and DUX4 expression were computed using the base package in R separately across LCL, myoblast, myotube and single cell myocyte samples following *z*-normalization within control matched pairs. Pearson correlations between the three DUX4 scores and the FSHD Lymphoblast score were computed using the base package in R, following *z*-normalization within each of the seven FSHD/control muscle biopsy studies considered.

In the case of the muscle biopsy dataset described by Wang *et al*. (49), a multivariate regression model was built for the FSHD Lymphoblast score and each of the three DUX4 scores to assess independent associations with the three histopathological and three MRI-based measures of disease activity paired with the RNA-seq samples.

### Study approval

Lymphoblastoid cell lines were characterized in Jacobsen *et al*. (41) and Jones *et al*. (42), where ethical permission is detailed. Primary FSHD and control myoblasts were described in Barro *et al*. (24) and ethical permission is contained therein.

## Supplementary Material

Banerji_et_al_HMG_2020_Figure_S1_ddaa053

Banerji_et_al_HMG_2020_Figure_S2_ddaa053

Banerji_et_al_HMG_2020_Figure_S3_ddaa053

Banerji_et_al_HMG_2020_Figure_S4_ddaa053

Banerji_et_al_HMG_2020_Table_S1__ddaa053

Banerji_et_al_HMG_2020_Table_S2_ddaa053
