# Supplementary material for: DUX4 expressing immortalized FSHD lymphoblastoid cells express genes elevated in FSHD muscle biopsies, correlating with the early stages of inflammation
Source: Hum Mol Genet. 2020 Apr 2;29(14):2285–99. doi: 10.1093/hmg/ddaa053 (PMC7424723; doi:10.1093/hmg/ddaa053)
Supplement: Banerji_et_al_HMG_2020_Figure_S1_ddaa053 [file banerji_et_al_hmg_2020_figure_s1_ddaa053.pdf]

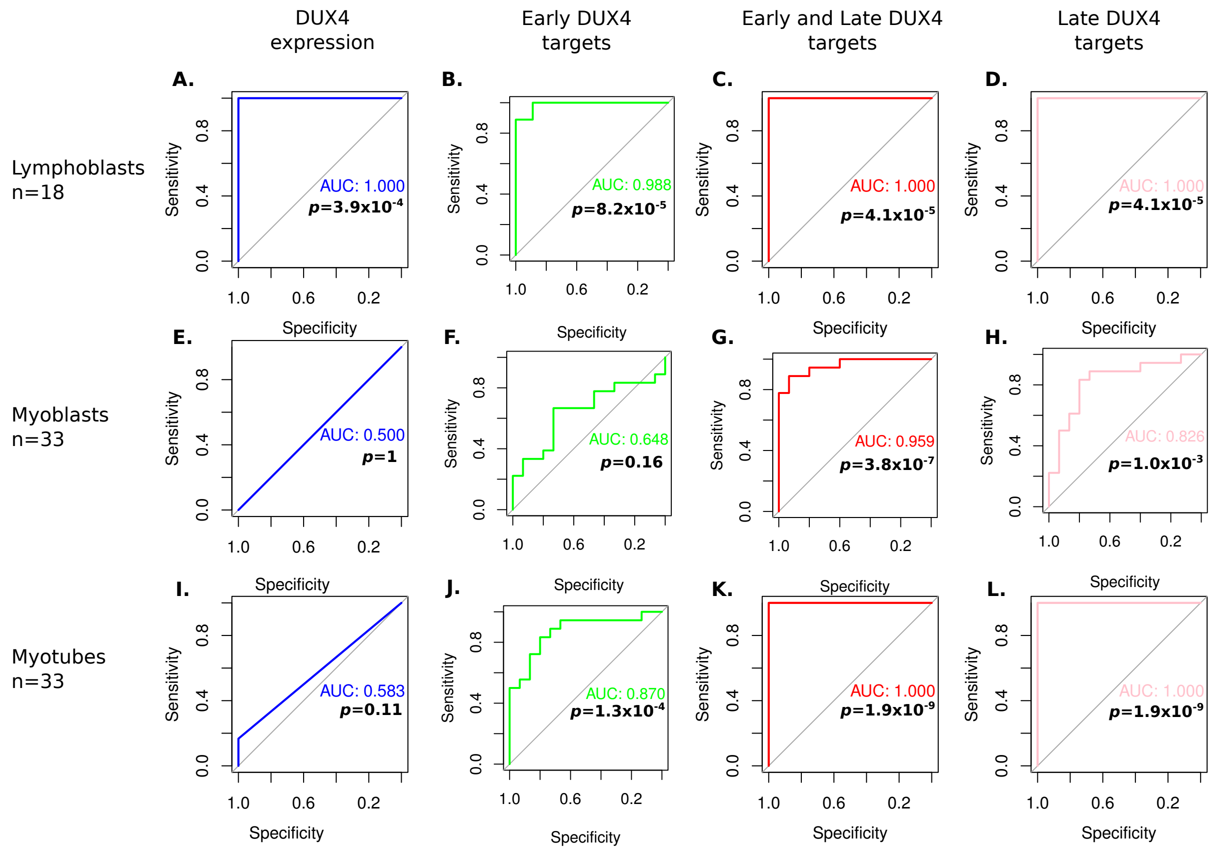

Figure S1: ***DUX4 and early and late DUX4 target gene expression identifies FSHD LCLs more robustly than FSHD myoblasts or myotubes.***

ROC curves display the discriminatory power of *DUX4* expression or expression of DUX4 target genes in patient derived LCLs (A-D), myoblasts (E-H) and differentiated myotubes (I-L), using the Choi et al. (1) (8 hour), Yao et al. (2) (24-48 hour) and Geng et al. (3) (24 hour) DUX4 target gene signatures. Early DUX4 targets are defined as those present exclusively in the Choi et al. DUX4 target gene set and absent from both Yao et al. and Geng et al. DUX4 target gene sets. Early and late DUX4 target genes are those present in both Choi et al., and either the Yao et al. or Geng et al. DUX4 target gene sets. Late DUX4 target genes are those absent from the Choi et al. DUX4 target gene set but present in either the Yao et al. or Geng et al. DUX4 target gene set. All scores were z-normalised within FSHD patient matched control group within cell type. AUC for each discriminator in each cell line is displayed alongside Wilcoxon p-values comparing the normalised biomarker value in FSHD samples vs controls. Only on LCLs are all 4 biomarkers perfect discriminators of FSHD status.

1 Choi, S.H., Gearhart, M.D., Cui, Z., Bosnakovski, D., Kim, M., Schennum, N. and Kyba, M. (2016) DUX4 recruits p300/CBP through its C-terminus and induces global H3K27 acetylation changes. *Nucleic Acids Res*, **44**, 5161-5173.

2 Yao, Z., Snider, L., Balog, J., Lemmers, R.J., Van Der Maarel, S.M., Tawil, R. and Tapscott, S.J. (2014) DUX4-induced gene expression is the major molecular signature in FSHD skeletal muscle. *Hum Mol Genet*, **23**, 5342-5352.

3 Geng, L.N., Yao, Z., Snider, L., Fong, A.P., Cech, J.N., Young, J.M., van der Maarel, S.M., Ruzzo, W.L., Gentleman, R.C., Tawil, R. *et al.* (2012) DUX4 activates germline genes, retroelements, and immune mediators: implications for facioscapulohumeral dystrophy. *Dev Cell*, **22**, 38-51.
