# Supplementary material for: DUX4 expressing immortalized FSHD lymphoblastoid cells express genes elevated in FSHD muscle biopsies, correlating with the early stages of inflammation
Source: Hum Mol Genet. 2020 Apr 2;29(14):2285–99. doi: 10.1093/hmg/ddaa053 (PMC7424723; doi:10.1093/hmg/ddaa053)
Supplement: Banerji_et_al_HMG_2020_Figure_S2_ddaa053 [file banerji_et_al_hmg_2020_figure_s2_ddaa053.pdf]

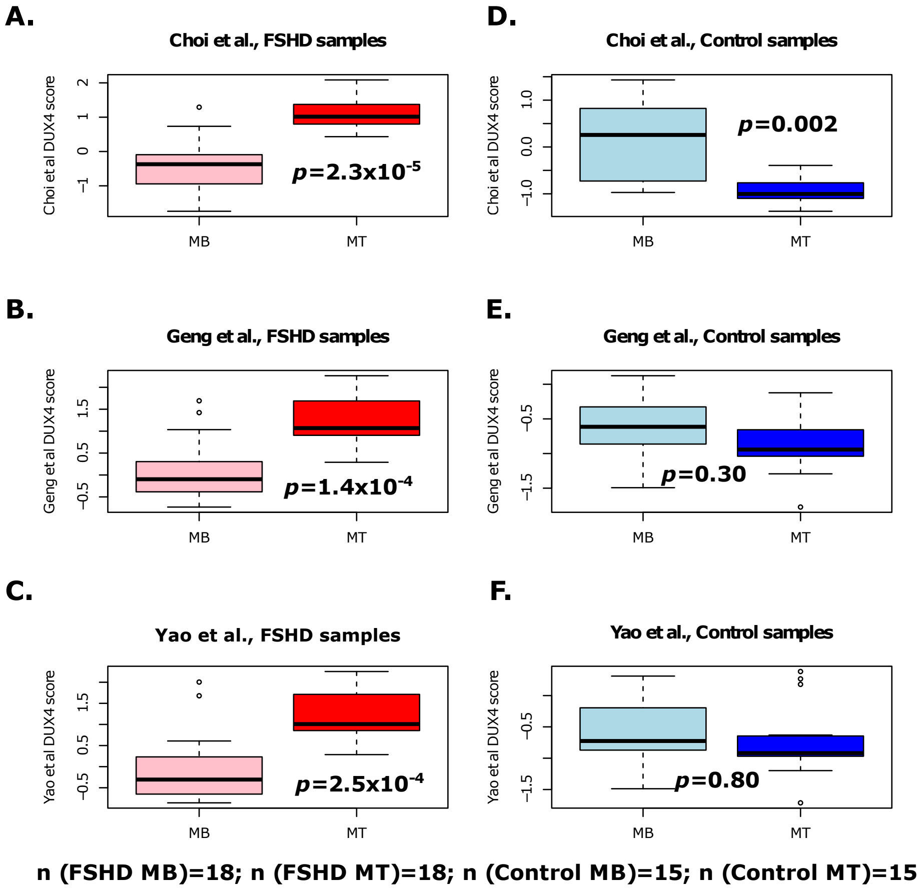

Figure S2: ***FSHD myotubes show up-regulation of early and late DUX4 target genes compared to matched myoblasts***

Boxplots demonstrate the Choi et al., (1) early (8 hour), and the Geng et al., (2) late (24 hour) and the Yao et al., (3) late (24-48 hour) DUX4 target gene signature (*z*-normalised within FSHD patient matched control group across both myoblasts and myotubes) in myoblasts and myotubes from FSHD samples (A-C) and control samples (D-F). In FSHD samples the three DUX4 target gene signatures were elevated on myotubes compared to matched myoblast samples. In control samples however, the Choi et al., (1) early DUX4 target gene signature was suppressed in myotubes compared to myoblasts while the two late DUX4 target gene signatures were unaltered. The box represents the interquartile range (IQR), with the median indicated by a line. Whiskers denote min (1.5*IQR, max (observed value)). ‘‘o’’ represents data points greater than 1.5 IQR from the median. Wilcoxon *U*-test *p*-values comparing myoblast to myotube samples are presented on each plot. MB: myoblast, MT: myotube.

(1) Choi, S.H., Gearhart, M.D., Cui, Z., Bosnakovski, D., Kim, M., Schennum, N. and Kyba, M. (2016) DUX4 recruits p300/CBP through its C-terminus and induces global H3K27 acetylation changes. *Nucleic Acids Res*, **44**, 5161-5173.
(2) Geng, L.N., Yao, Z., Snider, L., Fong, A.P., Cech, J.N., Young, J.M., van der Maarel, S.M., Ruzzo, W.L., Gentleman, R.C., Tawil, R. *et al.* (2012) DUX4 activates germline genes, retroelements, and immune mediators: implications for facioscapulohumeral dystrophy. *Dev Cell*, **22**, 38-51.
(3) Yao, Z., Snider, L., Balog, J., Lemmers, R.J., Van Der Maarel, S.M., Tawil, R. and Tapscott, S.J. (2014) DUX4-induced gene expression is the major molecular signature in FSHD skeletal muscle. *Hum Mol Genet*, **23**, 5342-5352.
