# Supplementary material for: DUX4 expressing immortalized FSHD lymphoblastoid cells express genes elevated in FSHD muscle biopsies, correlating with the early stages of inflammation
Source: Hum Mol Genet. 2020 Apr 2;29(14):2285–99. doi: 10.1093/hmg/ddaa053 (PMC7424723; doi:10.1093/hmg/ddaa053)
Supplement: Banerji_et_al_HMG_2020_Figure_S3_ddaa053 [file banerji_et_al_hmg_2020_figure_s3_ddaa053.pdf]

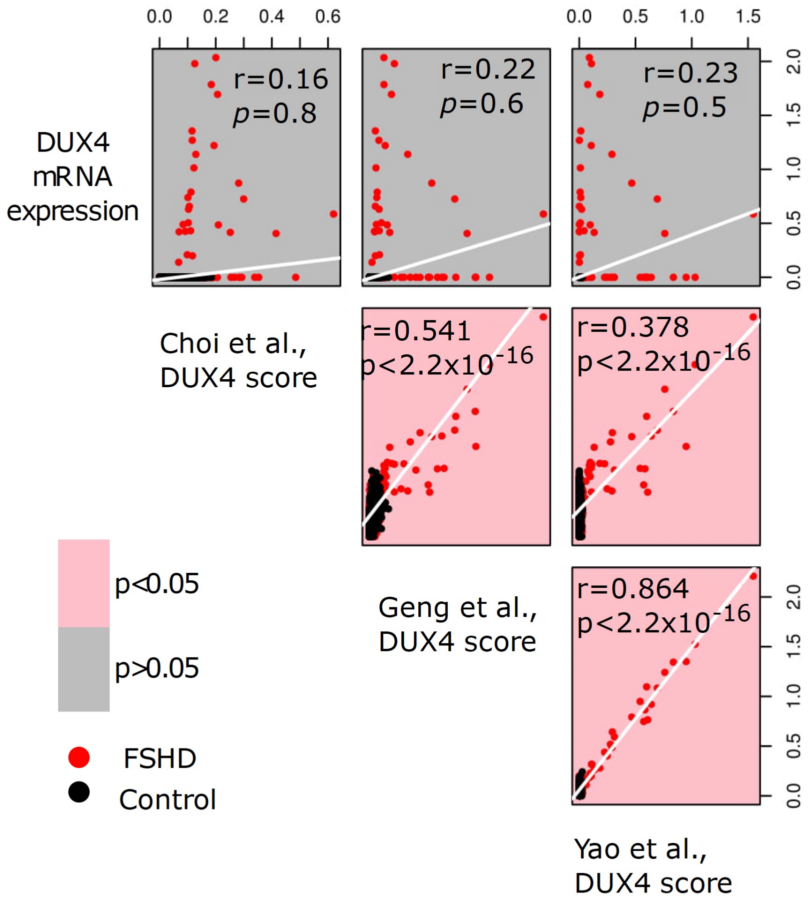

Figure S3: ***DUX4 displays burst-like expression in single cell RNA-seq of FSHD patient myocytes***
Scatter plots display *DUX4* expression, the Choi et al., (1) early (8 hour), the Geng et al., (2) late (24 hour) and the Yao et al., (3) late (24-48 hour) DUX4 target genes signature plotted against one another across the 7234 single myocyte samples from two FSHD1, two FSHD2 and two control patients described by van den Heuvel et al., 2019 (4). *DUX4* mRNA expression follows a burst-like expression pattern, attaining high values while DUX4 target gene expression is low, but *DUX4* expression then drops as DUX4 target gene expression rises. Pearson's *r* and associated *p*-value is given for each pairwise comparison. For *DUX4* mRNA, correlations are only made over the 27 *DUX4* expressing cells. Red dots correspond to FSHD samples, while black dots are controls. Plots denoting correlations reaching significance are coloured pink, whilst those not attaining significance are coloured grey.

(1) Choi, S.H., Gearhart, M.D., Cui, Z., Bosnakovski, D., Kim, M., Schennum, N. and Kyba, M. (2016) DUX4 recruits p300/CBP through its C-terminus and induces global H3K27 acetylation changes. *Nucleic Acids Res*, **44**, 5161-5173.
(2) Geng, L.N., Yao, Z., Snider, L., Fong, A.P., Cech, J.N., Young, J.M., van der Maarel, S.M., Ruzzo, W.L., Gentleman, R.C., Tawil, R. *et al.* (2012) DUX4 activates germline genes, retroelements, and immune mediators: implications for facioscapulohumeral dystrophy. *Dev Cell*, **22**, 38-51.
(3) Yao, Z., Snider, L., Balog, J., Lemmers, R.J., Van Der Maarel, S.M., Tawil, R. and Tapscott, S.J. (2014) DUX4-induced gene expression is the major molecular signature in FSHD skeletal muscle. *Hum Mol Genet*, **23**, 5342-5352.
(4) van den Heuvel, A., Mahfouz, A., Kloet, S.L., Balog, J., van Engelen, B.G.M., Tawil, R., Tapscott, S.J. and van der Maarel, S.M. (2019) Single-cell RNA sequencing in facioscapulohumeral muscular dystrophy disease etiology and development. *Hum Mol Genet*, **28**, 1064-1075.
