# Supplementary material for: DUX4 expressing immortalized FSHD lymphoblastoid cells express genes elevated in FSHD muscle biopsies, correlating with the early stages of inflammation
Source: Hum Mol Genet. 2020 Apr 2;29(14):2285–99. doi: 10.1093/hmg/ddaa053 (PMC7424723; doi:10.1093/hmg/ddaa053)
Supplement: Banerji_et_al_HMG_2020_Figure_S4_ddaa053 [file banerji_et_al_hmg_2020_figure_s4_ddaa053.pdf]

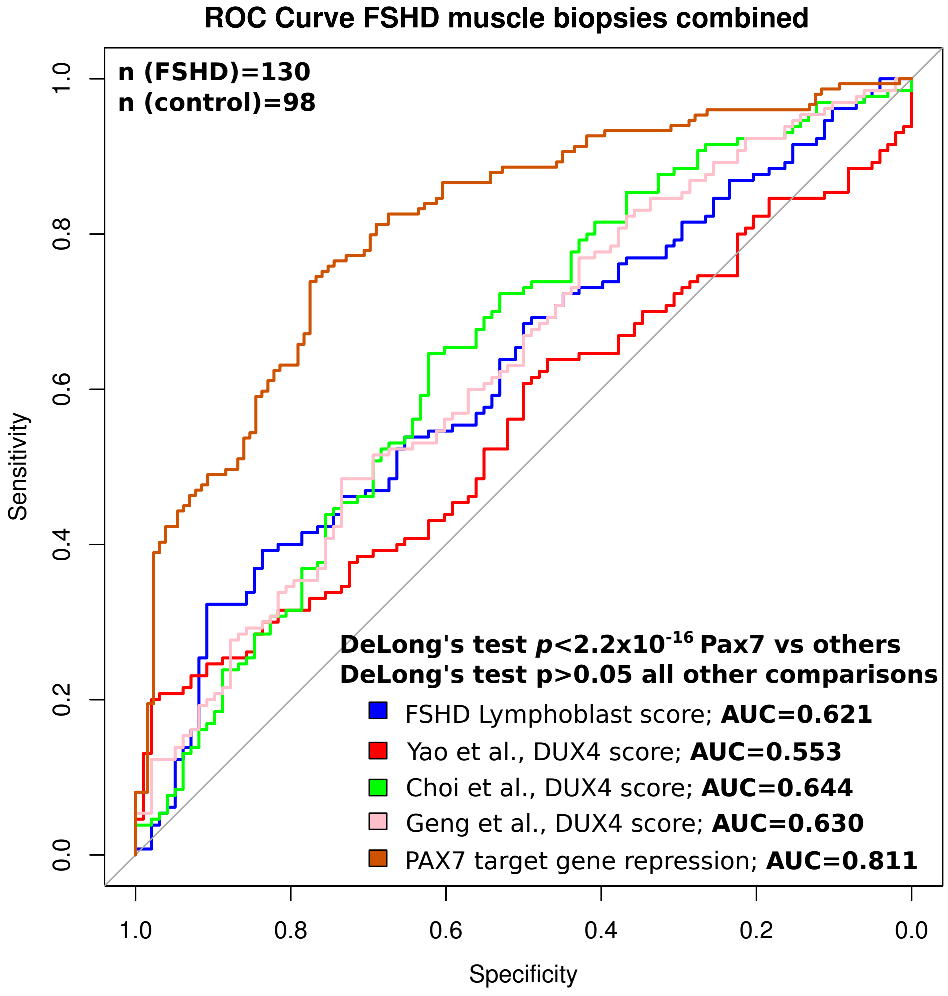

Figure S4: ***FSHD Lymphoblast score represents a moderately powered biomarker for FSHD muscle biopsies, equivalent to DUX4 target gene expression but inferior to PAX7 target gene repression.*** A ROC curve displays the discriminatory capacity of the FSHD Lymphoblast score (blue), the Choi et al., (1) early (8 hour) (green), the Yao et al., (2) late (24 - 48 hour) (red), and the Geng et al., (3) late (24 hour) (pink) DUX4 target gene signature and the PAX7 target gene repression score (brown) on all FSHD muscle biopsy datasets combined. The FSHD Lymphoblast score displays a discriminatory power similar to DUX4 target gene signatures, but inferior to PAX7 target gene repression. Each score was computed on each muscle biopsy sample and *z*-normalised within each of the seven independent studies before being pooled for ROC curve analysis. The AUC for each score is displayed alongside DeLong's test *p*-value comparing the discriminatory power of the biomarkers.

(1) Choi, S.H., Gearhart, M.D., Cui, Z., Bosnakovski, D., Kim, M., Schennum, N. and Kyba, M. (2016) DUX4 recruits p300/CBP through its C-terminus and induces global H3K27 acetylation changes. *Nucleic Acids Res*, **44**, 5161-5173.
(2) Yao, Z., Snider, L., Balog, J., Lemmers, R.J., Van Der Maarel, S.M., Tawil, R. and Tapscott, S.J. (2014) DUX4-induced gene expression is the major molecular signature in FSHD skeletal muscle. *Hum Mol Genet*, **23**, 5342-5352.
(3) Geng, L.N., Yao, Z., Snider, L., Fong, A.P., Cech, J.N., Young, J.M., van der Maarel, S.M., Ruzzo, W.L., Gentleman, R.C., Tawil, R. *et al.* (2012) DUX4 activates germline genes, retroelements, and immune mediators: implications for facioscapulohumeral dystrophy. *Dev Cell*, **22**, 38-51.
